# Assessment of aortic dilatation in Chinese children and adolescents with Turner syndrome: a single center experience

**DOI:** 10.1186/s12887-024-04783-2

**Published:** 2024-05-08

**Authors:** Wei Su, Longwei Sun, Zhuoguang Li, Xia Liu, Longjiang Zhang, Xiu Zhao, Shumin Fan, Boning Li, Ying Xie, Weiwei Xiao, Zhe Su

**Affiliations:** 1 Department of Endocrinology, Shenzhen Children’s Hospital, Shenzhen, China; 2 Department of Radiology, Shenzhen Children’s Hospital, Shenzhen, China; 3 Department of Ultrasound, Shenzhen Children’s Hospital, Shenzhen, China; 4 Department of Cardiology, Shenzhen Children’s Hospital, Shenzhen, China; 5 Department of Laboratory, Shenzhen Children’s Hospital, Shenzhen, China

**Keywords:** Aortic dilatation, Ascending aorta diameter, Turner syndrome, Turner syndrome-specific Z-score

## Abstract

**Background:**

Patients with Turner syndrome (TS) face an increased risk of developing aortic dilatation (AD), but diagnosing AD in children presents greater complexity compared to adults. This study aimed to investigate the application of various assessment indicators of AD in Chinese children and adolescents with TS.

**Methods:**

This study included TS patients admitted to Shenzhen Children’s Hospital from 2017 to 2022. Cardiovascular lesions were diagnosed by experienced radiologists. Patients without structural heart disease were divided into different body surface area groups, then the Chinese TS population Z-score (CHTSZ-score) of the ascending aorta was calculated and compared with other indicators such as aortic size index (ASI), ratio of the ascending to descending aortic diameter (A/D ratio), and TSZ-score (Quezada’s method).

**Results:**

A total of 115 TS patients were included, with an average age of 10.0 ± 3.7 years. The incidences of the three most serious cardiovascular complications were 9.6% (AD), 10.4% (coarctation of the aorta, CoA), and 7.0% (bicuspid aortic valve, BAV), respectively. The proportion of developing AD in TS patients aged ≥ 10 years was higher than that in those < 10 years old (16.6% vs. 1.8%, *P* = 0.009), and the proportion of patients with CoA or BAV who additionally exhibited AD was higher than those without these conditions (31.6% vs. 5.2%, *P* < 0.001). The ASI, A/D ratio, TSZ-score, and CHTSZ-score of the 11 patients with AD were 2.27 ± 0.40 cm/m^2^, 1.90 ± 0.37, 1.28 ± 1.08, and 3.07 ± 2.20, respectively. Among the AD patients, only 3 cases had a TSZ-score ≥ 2, and 2 cases had a TSZ-score ≥ 1. However, based on the assessment using the CHTSZ-score, 6 patients scored ≥ 2, and 5 patients scored ≥ 1. In contrast, the TSZ-score generally underestimated the aortic Z-scores in Chinese children with TS compared to the CHTSZ-score.

**Conclusions:**

The applicability of ASI and A/D ratio to children with TS is questionable, and racial differences can affect the assessment of TSZ-score in the Chinese population. Therefore, establishing the CHTSZ-score specifically tailored for Chinese children and adolescents is of paramount importance.

**Supplementary Information:**

The online version contains supplementary material available at 10.1186/s12887-024-04783-2.

## Introduction

Turner syndrome (TS), characterized by short stature and gonadal dysplasia, is the most prevalent sex chromosome abnormality in women, with an incidence of 1/2000–3000 live female births [1–3]. Congenital or acquired cardiovascular disease is very common and serves as the leading cause of early mortality among TS patients, of which aortic dilation (AD) and dissection represent the most lethal cardiovascular abnormalities [4, 5]. The onset of aortic dissection in patients with TS often precedes that observed in the general population, commonly manifesting during young to middle adulthood (usually between 20 and 40 years of age). Nonetheless, instances of aortic dissection have been documented in adolescents and even during childhood within this syndrome [5–8]. Major risk factors for aortic dissection in TS include AD, aortic coarctation (CoA), bicuspid aortic valve (BAV), hypertension, and others [3, 4]. Therefore, early detection through regular cardiac monitoring and imaging is crucial for timely intervention and management to mitigate the risks associated with aortic dissection in individuals with TS.

The ascending aorta is the most commonly affected site in TS patients compared to the sinus of Valsalva or the sino-tubular junction [5]. Various indicators are employed for evaluating AD, such as the aortic size index (ASI), ratio of the ascending to descending aortic diameter (A/D ratio), and the Turner syndrome-specific Z-score (TSZ-score) [5, 9], each with its own advantages and disadvantages. Cardiovascular imaging provides relatively clear criteria for diagnosing AD in adults: an ascending aorta ASI ≥ 2.0 cm/m^2^, A/D ratio ≥ 1.5, or ascending aorta TSZ-score ≥ 2 is defined as dilatation in adults [9, 10]. However, these standards remain controversial in pediatric clinical practice, especially regarding their applicability in Chinese children, which has not yet been verified. Therefore, by conducting a retrospective analysis of the cardiovascular characteristics of Chinese children and adolescents with TS, with a particular emphasis on exploring the practical value of various cardiovascular assessment indicators in the evaluation of AD, this study aimed to provide experience for future clinical practice.

## Materials and methods

### Study participants

In this study, a total of 115 patients with TS admitted to Shenzhen Children’s Hospital from September 2017 to November 2022 were included. All participants underwent a systematic evaluation according to the guidelines of the 2016 Cincinnati International Turner Syndrome Conference [3], and our main focus in this study was the analysis of cardiovascular examinations and their results.

#### Cardiovascular imaging tests

Cardiovascular computed tomography angiography (CCTA) was performed using a 64-multidetector computed tomography scanner (General Electric Optima 680, GE Healthcare, Boston, MA, USA), with image scanning from the thoracic inlet to the most caudal margin of the heart. The maximum diameter of the ascending aorta was measured from inner edge to inner edge in the axial plane during the diastolic phase, while the diameter of the descending aorta was measured at the level of the diaphragm (mm). CCTA examination results of enrolled patients underwent independent analysis in a blinded manner by two cardiovascular radiologists, each with equivalent qualifications and over 10 years of experience. One doctor repeated the analysis one year later under blinded conditions for validation. The external and internal agreement of the CCTA results was assessed using the diameter of the ascending aorta as a reference index. In the initial judgment, dilation was defined by a Z-score ≥ 2, calculated based on the normal values of aortic diameters in children and adolescents provided by the University Children’s Hospital Zurich (Switzerland) [11].

#### Assessment of aortic dilatation

(1) The ASI of the ascending aorta was calculated using the Haycock body surface area (BSA) equation [aortic diameter (cm) / BSA (m^2^)] [12]. (2) The A/D ratio of the patients was also calculated. (3) The TSZ-score of the ascending aorta was calculated using the calculator provided by Quezada (http://www.parameterz.com/refs/quezada-ajmg-2015) [13]. (4) Given that the TSZ-score system was established based on the Caucasian race, there are race-related disparities in practical applications. Thus, we introduced the concept of the Chinese TS population Z-score (CHTSZ-score) in this study. Patients without structural heart disease were categorized into four groups based on their BSA (≥ 0.50 and < 0.75 m^2^, ≥ 0.75 and < 1.00 m^2^, ≥ 1.00 and < 1.25 m^2^, ≥ 1.25 and < 1.50 m^2^), with their ascending aorta diameter serving as a reference for TS patients.

### Statistical analysis

Statistical analysis was performed using the SPSS software (version 25.0; SPSS Inc. Chicago, IL, USA). The normality of the data was assessed using the Shapiro-Wilk test. Continuous variables were presented as mean ± standard deviation or median (interquartile range), as appropriate. The independent sample t-test was used to compare two groups for continuous variables with normal distribution, while the chi-square test was used for categorical variables. Kendall’s coordination coefficient was used to assess the external and internal agreement, with a W value closer to 1 indicating strong agreement and closer to 0 indicating weak agreement. Pearson or Spearman correlation analysis was performed for correlation analysis. The sensitivity and specificity of each indicator for AD were calculated, and the Youden index was determined as: (sensitivity + specificity) – 1. Statistical significance was set at *P* < 0.05 (two-tailed).

## Results

### Clinical manifestations

A total of 115 patients with TS were enrolled in this study, with an average age at assessment of 10.0 ± 3.7 years. The TS monosomy (45, X) karyotype was observed in 43.5% (*n* = 50) of patients, while 56.5% (*n* = 65) had a non-TS monosomy (non-45, X) karyotype. Further details can be found in Supplementary Table S1. Clinical manifestations included short stature (97.4%), scoliosis (55.7%), kidney deformities (horseshoe kidney, duplication of kidneys, or hydronephrosis) (19.1%), otitis media (16.5%), impaired glucose tolerance (8.7%), strabismus (8.7%), autoimmune thyroid diseases (7.8%), and hearing loss (7.8%).

**Fig. 1:**
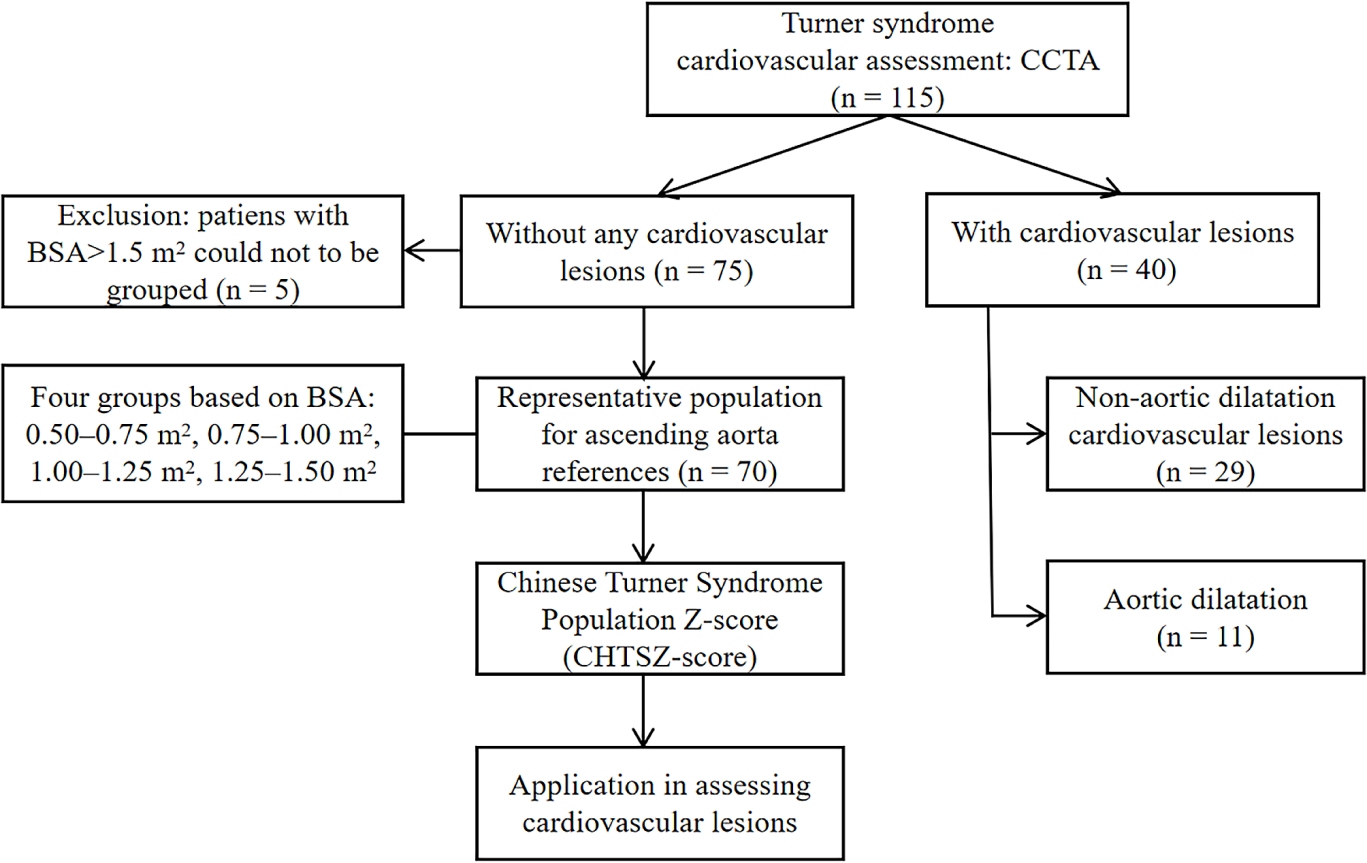
Flow chart of the cardiovascular assessment process

All CCTA results were comprehensively reviewed by two cardiovascular radiologists and cardiovascular lesions were diagnosed. See Fig. 1 for details. The Kendall’s coordination coefficient W values of internal and external agreement were 0.999 and 0.998, respectively (both *P* < 0.001), which indicates strong consistency. Among them, the incidences of the three most serious cardiovascular complications were 9.6% (11/115) for AD, 10.4% (12/115) for CoA, and 7.0% (8/115) for BAV, respectively. Other cardiovascular lesions included partial anomalous pulmonary venous return (13.9%), persistent left superior vena cava (4.3%), patent ductus arteriosus (1.7%), and atrial septal defect (0.8%).

### Involvement of AD and comparison of different assessment criteria

**Fig. 2:**
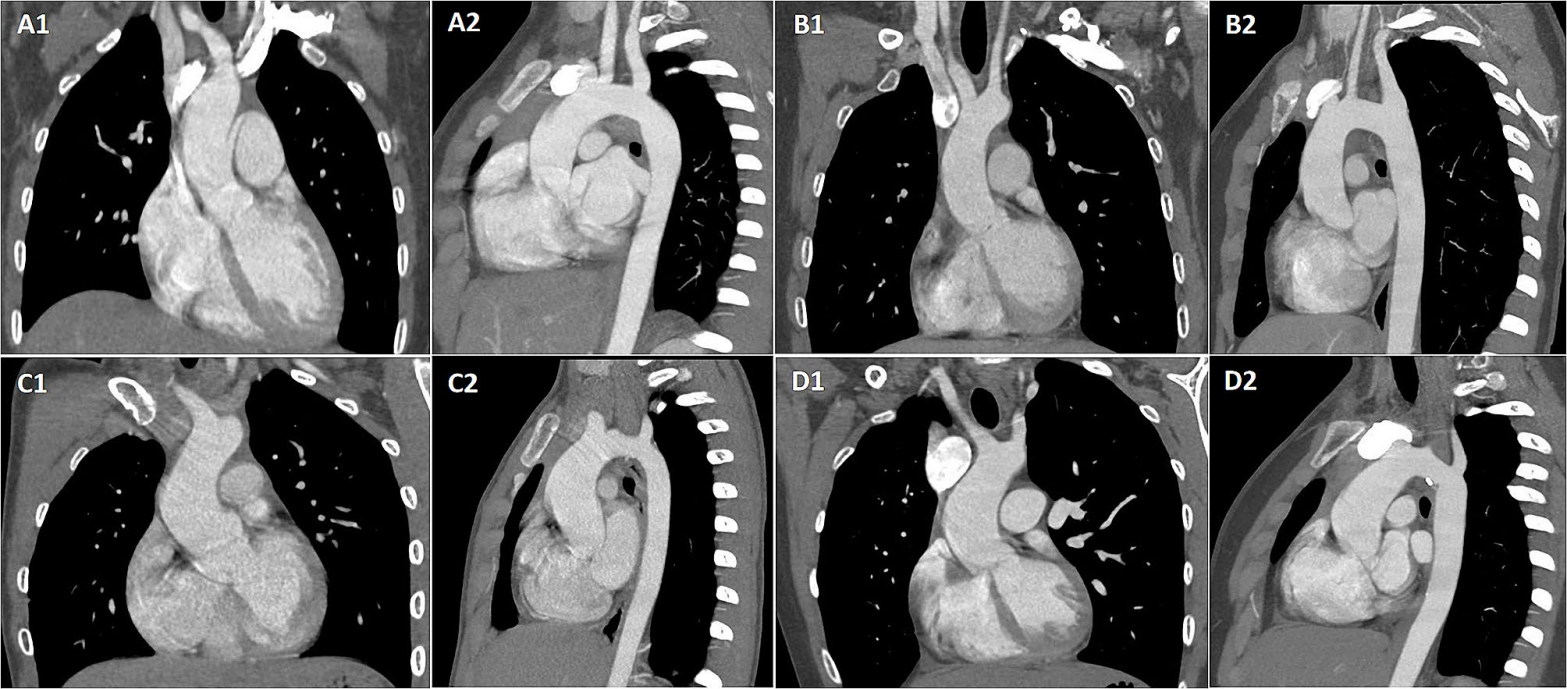
Representative images of dilated aorta in patients with Turner syndrome. Note: 1 represents axial direction, 2 represents sagittal direction. Patient A: 10.5 years old, ASI: 2.10 cm/m^2^, A/D ratio: 1.93, TSZ-score: 0.30, CHTSZ-score: 1.39; Patient B: 16.0 years old, ASI: 1.80 cm/m^2^, A/D ratio: 1.35, TSZ-score: 0.27, CHTSZ-score: 1.35; Patient C: 11.0 years old, ASI: 2.99 cm/m^2^, A/D ratio: 2.45, TSZ-score: 2.42, CHTSZ-score: 7.95; Patient D: 13.5 years old, ASI: 2.44 cm/m^2^, A/D ratio: 1.72, TSZ-score: 2.40, CHTSZ-score: 2.91

In this study, a total of 11 patients with TS were initially defined as AD by experienced radiologists. Their average age was 13.2 ± 2.7 years, and the diameter of the ascending aorta reached 25.9 ± 4.0 mm. The proportion of developing AD in TS patients aged ≥ 10 years was higher than that in those < 10 years old (10/60, 16.6% vs. 1/55, 1.8%, *P* = 0.009). Furthermore, the proportion of patients with BAV or CoA who additionally exhibited AD was higher than those without these conditions (6/19, 31.6% vs. 5/96, 5.2%, *P* < 0.001). Some representative CCTA images of patients with AD are shown in Fig. 2.

#### ASI assessment

There were 22 cases with ASI ≥ 2.0 cm/m^2^ at an average age of 8.4 ± 3.6 years. Among them, the incidence of AD, CoA and BAV were 31.8%, 27.2%, and 13.6%, respectively. The ASI of AD patients (2.27 ± 0.40 cm/m^2^) was significantly higher than that of patients without AD (1.68 ± 0.34 cm/m^2^) (*P* < 0.001). A significant negative correlation was observed between ASI and age (*P* < 0.001), indicating that applying adult diagnostic criteria for ASI (≥ 2.0 cm/m^2^) in children could result in overdiagnosis. See Supplementary Figure S1 for details.

#### A/D ratio assessment

There were 31 cases with A/D ratio ≥ 1.5 at an average age of 10.3 ± 3.2 years. Among them, the incidence of AD, CoA and BAV were 32.2%, 22.5%, and 12.9%, respectively. The A/D ratio of AD patients (1.90 ± 0.37) was significantly higher than that of patients without AD (1.38 ± 0.18)(*P* < 0.001).

#### TSZ-score and CHTSZ-score assessment

**Fig. 3:**
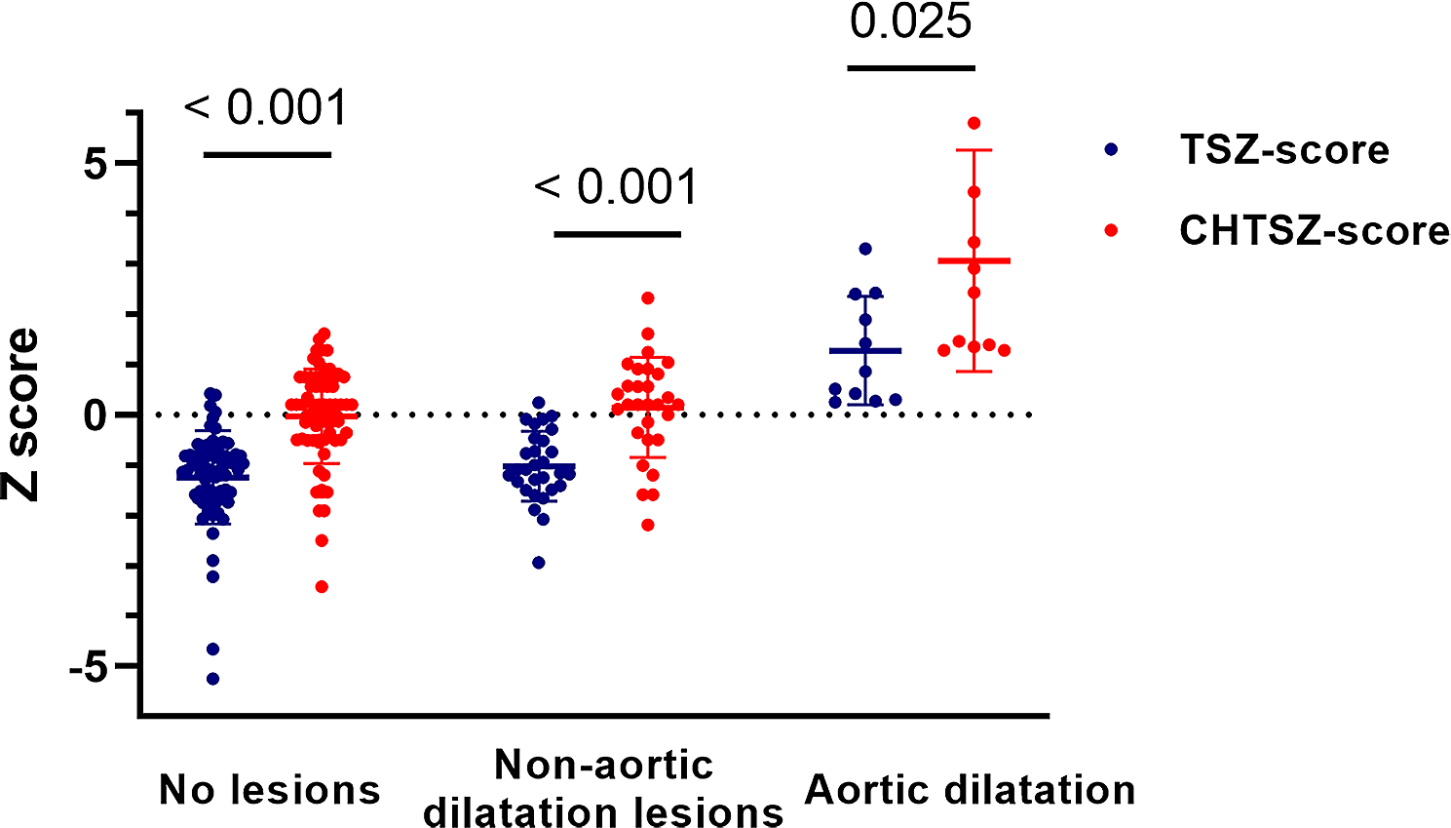
Comparison of TSZ-score and CHTSZ-score assessment in Chinese children with TS

Based on the TSZ-score calculator provided by Quezada, although the TSZ-score of AD patients (1.28 ± 1.08) was higher than that of patients without AD (-1.18 ± 0.85) (*P* < 0.001), there were only 3 patients diagnosed with AD had a TSZ-score ≥ 2, and 2 patients had a TSZ-score ≥ 1. As for the CHTSZ-score assessment, the average CHTSZ-score of the AD patients was 3.07 ± 2.20, with 6 patients being ≥ 2 and 5 patients being ≥ 1. In comparison to the CHTSZ-score, the TSZ-score generally underestimated the Z scores of the aorta in Chinese children with TS, whether in non-cardiovascular lesions, non-AD cardiovascular lesions, or AD. See Fig. 3 and Supplementary Table S2 for details.

In the sensitivity and specificity analyses, when a TSZ-score of ≥ 2 was used to diagnose patients with AD, only 27.3% (3/11) were diagnosed with high specificity (100%) and low sensitivity. The sensitivity of the TSZ-score was lower compared to ASI (7/11, 63.6%), A/D ratio ≥ 1.5 (10/11, 90.9%) and CHTSZ-score ≥ 2 (6/11, 54.5%). The Youden indices for ASI, A/D ratio, TSZ-score, and CHTSZ-score of AD assessment were 0.489, 0.707, 0.273, and 0.535, respectively.

## Discussion

This is a single-center study focusing on comprehensive cardiovascular assessments of Chinese children and adolescents with TS according to the 2016 Cincinnati clinical practice guidelines. Among the young individuals with TS in this study, the incidence of the three most serious cardiovascular complications (namely, AD, CoA and BAV) was all observed to be close to 10%. Since CoA and BAV are primarily congenital cardiovascular diseases, their detection should raise suspicion of TS even in neonates [2, 4, 14]. The lifetime incidence rates of CoA and BAV remained relatively stable, with rates of 10.4% and 7.0%, respectively, observed in our study, figures that are closely related to those reported in adult TS patients [2]. However, AD often manifests as an acquired vascular condition, with its incidence potentially increasing with age. In our study, the prevalence of AD among patients aged 10 years and older was significantly higher compared to those younger patients, aligning with rates reported in adult patients with TS (16.2–22%) [4, 15, 16]. A French longitudinal study, including 197 adults with TS, also found that the incidence of AD increased from 16.2 to 17.5% after 5 years of follow-up [17]. Recognized risk factors for AD mainly include other structural abnormalities (such as CoA and BAV) or physiological risk factors (like hypertension) [18]. Notably, in our study, the proportion of patients with CoA or BAV who additionally exhibited AD was higher than those without these conditions. Therefore, as mentioned in the guidelines, patients with TS with CoA or BAV may require more frequent cardiovascular monitoring.

To date, only a limited number of studies have concentrated on cardiovascular assessment in children and adolescents with TS. Due to the ongoing rapid growth and development of vascular morphology and body size in these patients, the evaluation of AD in children and adolescents needs to be adjusted based on BSA. This raises a pertinent question regarding the reference set: Previous studies have found that when healthy individuals are used as a reference, the incidence of AD by transthoracic echocardiography and magnetic resonance arteriography appears significantly higher than the theoretical value, reaching 30% and 16%, respectively [19, 20]. Conversely, when using TS patients without cardiovascular lesions as a reference, the incidence of AD (TSZ-score ≥ 2) was notably much lower (2%) [19]. In clinical practice, the evaluation of AD often demands the consideration of multiple parameters, such as ASI, A/D ratio, and TSZ-score. However, unlike adults, children lack clear diagnostic thresholds, thus leading to clinical dilemmas.

It is widely recognized that short stature is the most common clinical characteristic of patients with TS, necessitating the adjustment of aortic size based on BSA, commonly referred to as ASI. In adults, ASI is commonly used for medical and surgical decision-making, for instance, Davis et al. employed ASI to stratify disease risk for aneurysms, which has important clinical implications [21]. However, ASI has rarely been studied in children. In the present study, the ASI of AD patients was significantly higher than that of patients without AD, and the sensitivity of the ASI threshold (≥ 2.0 cm/m^2^) for diagnosing AD was 63.6%. However, it’s important to remain vigilant regarding the significant negative correlation observed between ASI and age. Younger children often exhibited higher ASI values, implying a potential risk of overdiagnosis if adult ASI diagnostic criteria were indiscriminately applied to pediatric patients. Furthermore, the A/D ratio proves beneficial in standardizing the ascending aorta to the presumed internal criteria for the descending aorta of each person, defining a dilatation ratio of ≥ 1.5 [22]. In our study, the sensitivity of the A/D threshold ≥ 1.5 was 90.9%, indicating it as the most sensitive parameter for assessing AD. However, this parameter overlooks the possibility of abnormalities in the descending aorta. In summary, while ASI and A/D ratio demonstrate good sensitivity in assessing AD in our study, their inherent shortcomings restrict their usage in the pediatric population, hence they are not recommended by pediatric TS guidelines.

Due to the necessity for scaling based on body size, absolute measurements of the aorta in pediatric patients are converted to Z-scores using BSA. However, concerns have been raised regarding the short stature and small BSA, as well as differences in somatic growth trajectories in patients with TS. Therefore, Quezada et al. recently introduced the TSZ-score formula by using healthy girls and women with TS as reference populations (based on 481 individuals with TS aged 3–70 years who underwent echocardiography examinations) [13]. Since ascending aortic ASI is age-dependent for individuals under 15 years, the TSZ-score may aid decision-making to some extent, leading to its gradual recommendation by guidelines [3, 22, 23]. However, due to racial differences (with East Asians commonly exhibiting lower BSA than Caucasians), the TSZ-score tends to underestimate aortic lesions in the Chinese TS population. In our study, the sensitivity of the TSZ-score (≥ 2.0) was found to be only 27.3% among AD patients. This is noteworthy because a missed diagnosis could result in an underestimation of AD and increase the potential risk of sudden death associated with aortic dissection. Enhancing the sensitivity of diagnosing AD in TS patients is crucial from the perspective of life safety. Hence, we advocate for the establishment of a TSZ-score reference specifically tailored for young Chinese TS patients. Based on the CHTSZ-score, among the AD patients, 6 cases scored ≥ 2, and 5 cases scored ≥ 1. However, due to the limited sample size in this study, the developed CHTSZ-score did not yield satisfactory results, highlighting the necessity for further refinement in our future research.

Several limitations of our study should be acknowledged. Firstly, there was currently no reference available for assessing the ascending aorta in Chinese children and adolescents with TS, potentially rendering our initial diagnosis of AD contentious. Additionally, as a single-center study conducted in China, the limited number of patients with TS without cardiovascular lesions was insufficient to draw a definite conclusion. Therefore, multi-center cooperation would be essential to establish a comprehensive reference for young Chinese patients with TS.

## Conclusions

Our study represents the initial reporting of cardiovascular assessment findings among children and adolescents with TS in a single center in China. Notably, individuals with TS face an elevated risk of developing AD, with age and other cardiovascular anomalies such as CoA or BAV serving as significant risk factors. The applicability of ASI and A/D ratio to children with TS is questionable, and racial differences can affect the assessment of TSZ-score in the Chinese population. Therefore, establishing CHTSZ-score specifically tailored for Chinese children and adolescents is of paramount importance.

### Electronic supplementary material

Below is the link to the electronic supplementary material.

Supplementary Material 1

## Data Availability

The datasets used and/or analyzed during the current study are available from the corresponding author upon reasonable request.

## Abbreviations

TS: Turner syndrome
AD: Aortic dilatation
ASI: Aortic size index
A/D ratio: Ratio of the ascending to descending aortic diameter
TSZ-score: Turner syndrome-specific Z-score
CHTSZ-score: Chinese Turner syndrome population Z-score
CoA: Aortic coarctation
BAV: Bicuspid aortic valve
BSA: Body surface area
CCTA: Cardiovascular computed tomography angiography
