# Supplementary material for: Assessment of aortic dilatation in Chinese children and adolescents with Turner syndrome: a single center experience
Source: BMC Pediatr. 2024 May 8;24:317. doi: 10.1186/s12887-024-04783-2 (PMC11077734; doi:10.1186/s12887-024-04783-2)
Supplement: Supplementary file 1 — Supplementary Material 1 [file 12887_2024_4783_MOESM1_ESM.docx]

**Supplementary Table S1.** Chromosomal karyotypes of the 115 patients with TS

| **Karyotype** | **Case** | **Percentage** | **Total** |
| --- | --- | --- | --- |
| **Monosomy** |  |  | 43.5% |
| 45, X | 50 | 43.5% |  |
| **Mosaic** |  |  | 12.2% |
| 45, X/46, XX | 8 | 7.0% |  |
| 45, X/47, XXX | 2 | 1.7% |  |
| 45, X, inv(q) | 4 | 3.5% |  |
| **Mosaic with variant** |  |  | 32.2% |
| 45, X /46, Xi(Xq) | 10 | 8.7% |  |
| 45, X /46, X, +Mar | 10 | 8.7% |  |
| 45, X /46, X, +r(?) | 4 | 3.5% |  |
| 45, X /46, X, del(X) | 5 | 4.3% |  |
| 45, X /47, XYY/46, XY | 1 | 0.9% |  |
| 45,X /46, XY | 5 | 4.3% |  |
| 45, X /47, X, i(Y), i(Y)/46, X, i(Y)  45, X /46, Xi(Xq)/ 46, X, del(X) | 1  1 | 0.9%  0.9% |  |
| **Variant** |  |  | 12.2% |
| 46, Xi(Xq) | 8 | 7.0% |  |
| 46, X, del(X) | 6 | 5.2% |  |

**Supplementary Table S2.** CHTSZ-score and TSZ-score assessment in 11 TS patients with AD based on different BSA group

| **BSA (m^2^)** | **Ascending aortic diameter (mm)** | **AD patients (n=11)** | |
| --- | --- | --- | --- |
|  |  | **CHTSZ-score** | **TSZ-score** |
| 0.50–0.75 (n=19) | 11.99 ± 2.68 | / | / |
| 0.75–1.00 (n=22) | 15.71 ± 1.42 | 4.43  7.95 | 0.86  2.42 |
| 1.00–1.25 (n=18) | 17.43 ± 2.79 | 1.39 | 0.30 |
|  |  | 1.46 | 0.25 |
|  |  | 3.43 | 1.89 |
|  |  | 5.80 | 3.30 |
| 1.25–1.50 (n=11) | 18.54 ± 4.28 | 1.28 | 0.42 |
|  |  | 1.28 | 0.51 |
|  |  | 1.35 | 0.27 |
|  |  | 2.44 | 1.42 |
|  |  | 2.91 | 2.40 |

**Supplementary Table S3.** Sensitivity and specificity of different indicators of aortic dilation

| **Indicator** | **Sensitivity** | **Specificity** | **Youden index** |
| --- | --- | --- | --- |
| ASI (≥ 2 cm/m^2^) | 63.6% (7/11) | 85.6% (89/104) | 0.489 |
| A/D ratio (≥ 1.5) | 90.9% (10/11) | 79.8% (83/104) | 0.707 |
| TSZ-score (≥ 2) | 27.3% (3/11) | 100% (104/104) | 0.273 |
| CHTSZ-score (≥ 2) | 54.5% (6/11) | 99.0% (98/99)# | 0.535 |

Note: # 5 patiens with BSA > 1.5 m^2^ were excluded.


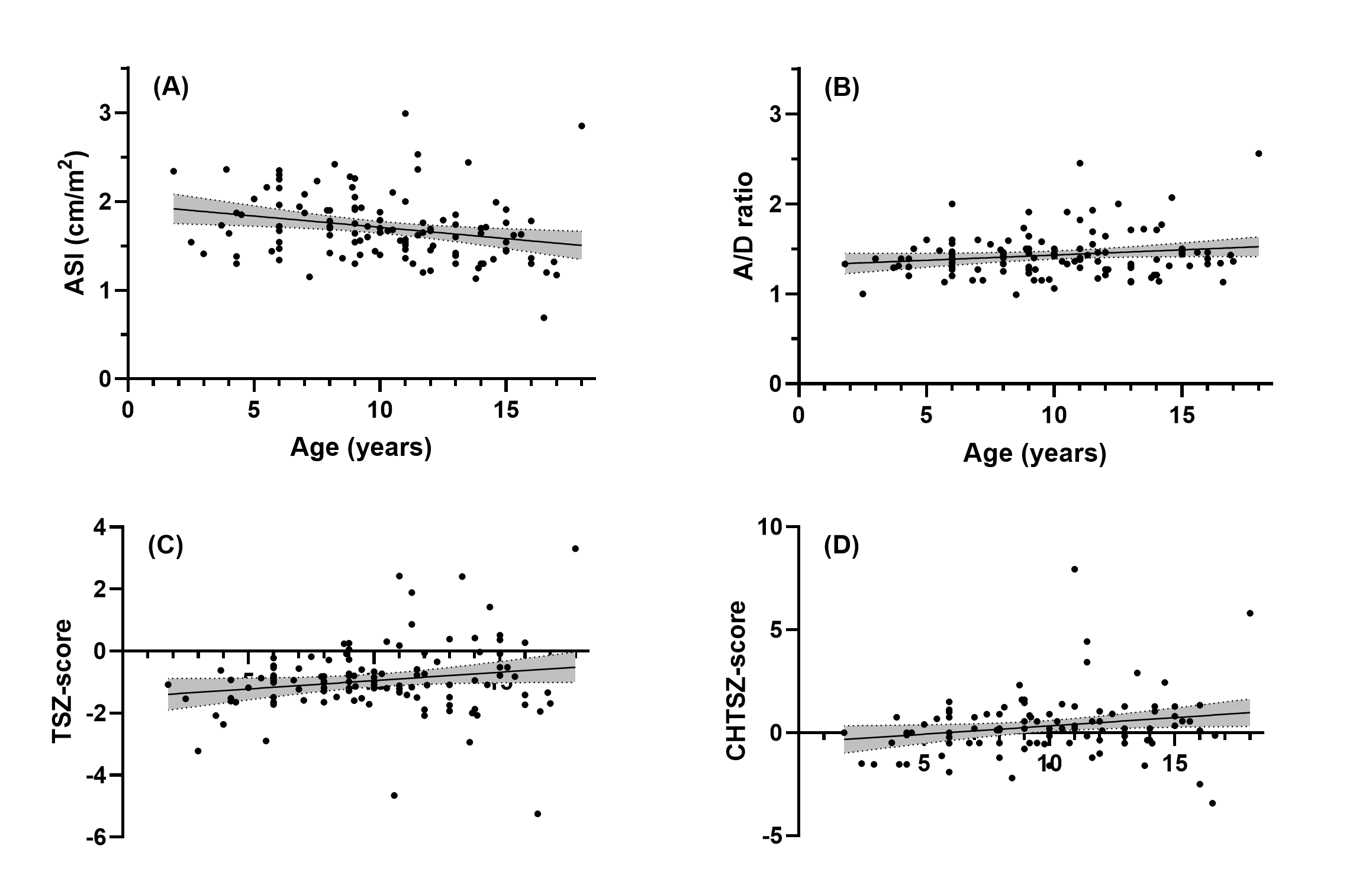


**Supplementary Figure S1.** Correlation analysis between age and different assessment indicators of aortic dilation

Note: Spearman correlation analysis was used for non-normally distributed data. Among them, the *P* values of A–D correlation analysis were 0.001, 0.384, 0.159, and 0.076, respectively.
